# Trends in pulmonary embolism in patients infected with HIV during the combination antiretroviral therapy era in Spain: A nationwide population-based study

**DOI:** 10.1038/s41598-018-29739-2

**Published:** 2018-08-14

**Authors:** Alejandro Alvaro-Meca, Pablo Ryan, Dariela Micheloud, Angel De Miguel, Juan Berenguer, Salvador Resino

**Affiliations:** 1 Departamento de Medicina Preventiva y Salud Pública, Facultad de Ciencias de la Salud, Universidad Rey Juan Carlos, Alcorcón, Madrid Spain; 2 Servicio de Medicina Interna, Hospital Universitario Infanta Leonor, Madrid, Spain; 3 Servicio de Urgencias, Hospital General Universitario “Gregorio Marañón”, Madrid, Spain; 4 Unidad de Enfermedades Infecciosas/VIH; Hospital General Universitario “Gregorio Marañón”, Madrid, Spain; 5 Instituto de Investigación Sanitaria Gregorio Marañón (IiSGM), Madrid, Spain; 6 Unidad de Infección Viral e Inmunidad, Centro Nacional de Microbiología, Instituto de Salud Carlos III, Majadahonda, Madrid Spain

## Abstract

Chronic infections are a major factor in the development of pulmonary embolism (PE). We aimed to evaluate the trends of PE-related hospitalizations and PE-related deaths in people living with HIV (PLWH) during the era of combination antiretroviral therapy (cART) through a retrospective study in Spain. Data were collected from the Minimum Basic Data Set (MBDS) between 1997 and 2013. The study period was fragmented into four calendar periods (1997–1999, 2000–2003, 2004–2007, and 2008–2013). The rate of PE-related hospitalizations remained stable in PLWH (*P* = 0.361). HIV-monoinfected patients had a higher incidence than HIV/HCV-coinfected patients during all follow-up [(98.7 (95%CI = 92.2; 105.1); *P* < 0.001], but PE incidence decreased in HIV-monoinfected patients (*P* < 0.001) and increased in HIV/HCV-coinfected patients (*P* < 0.001). Concretely, the rate of PE-related hospitalizations decreased significantly in patients monoinfected with HIV [from 203.6 (95%CI = 175.7; 231.6) events per 100,000 patient-years in 1997–1999 to 74.3 (95%CI = 66.1; 82.3) in 2008–2013; *P* < 0.001], while patients coinfected with HIV/HCV had a significant increase [from 16.3 (95%CI = 11; 21.6) in 1997–1999 to 53.3 (95%CI = 45.9; 60.6) in 2008–2013; *P* < 0.001]. The mortality rate of PE-related hospitalizations showed a similar trend as PE incidence. In conclusion, the epidemiological trends of PE in PLWH changed during the cART era, with decreases in incidence and mortality in HIV-monoinfected and increases in both variables in patients coinfected with HIV/HCV.

## Introduction

Hepatitis C virus (HCV) and human immunodeficiency virus (HIV) infections overlap in both modes of transmission and affected populations. Around 2.3 million people living with HIV are coinfected with HCV globally, and of these, 1.3 million are injection drug users (IDUs)^1^. In Spain, the current prevalence of HIV-infected patients with HCV antibodies and active HCV infection is 37.7% and 22.1%, respectively. However, this is significantly lower than the prevalences recorded in 2002 and 2009^2^.

HIV infection is currently a manageable chronic disease in high-income countries since the introduction of combination antiretroviral therapy (cART)^3^. Patients infected with HIV are living long enough to face significant morbidity from chronic illnesses such as cardiovascular disease^4,5^. Moreover, chronic hepatitis C has become significant comorbidity in HIV-infected subjects with HCV infection and seems to have a negative impact on the clinical course of HIV-infected patients, since it increases both HIV-associated mortality and overall mortality^6,7^.

Pulmonary embolism (PE) is the most clinical severe presentation of thromboembolic disease, and its incidence varies from country to country, with Spain having around 20–35 events per 100,000 person-year in the general population^8^. PE causes significant morbidity and mortality and a substantial economic burden in developed countries^9,10^. The risk factors associated with PE seem to be a combination of patient-specific factors (chronic diseases, anomalies of hemostasis, age, etc.) and precipitating factors (catheterization, surgery, acute pathologies, acute venous stasis, chronic infections, etc.)^11^. Chronic infections can act as a trigger factor by inducing immune activation, synthesis of hepatic proteins associated with inflammation, and modification of the fibrinolysis and coagulation pathways^11^.

There is little knowledge of PE epidemiology in people living with HIV (PLWH). The incidence of venous thromboembolism in PLWH is higher than in non-HIV patients^12,13^. Malek *et al*. found that HIV-infected individuals are more likely to have clinically detected PE compared to non-HIV-infected individuals^14^. Regarding chronic hepatitis C, in a recent meta-analysis, HCV-infected patients showed a significantly increased risk of venous thromboembolism^15^, but no significant association was found for PE. Moreover, HIV/HCV-coinfected patients, when compared to HIV- or HCV-monoinfected subjects, are a subgroup that may differ regarding inflammatory profile and risk factor distribution^16^, which might influence the risk of PE differently.

We aimed to evaluate the epidemiological trends of hospitalizations related to PE and PE-related deaths in PLWH during the cART era in Spain.

## Results

### Characteristics of the study population

Overall, 267,507 patients discharged with a PE diagnosis were found from 1997 to 2013 in Spanish hospitals. Among those patients, 1,356 were HIV-infected subjects (899 monoinfected with HIV and 457 coinfected with HIV/HCV) (Table 1). HIV/HCV-coinfected subjects had higher percentages of tobacco abuse (*P* < 0.001) and chronic pulmonary disease (*P* < 0.001) than HIV-monoinfected subjects.

**Table 1:** Summary of epidemiological and clinical data of subjects with PE-related hospital admissions in Spain (1997 to 2013).

	HIV-infected	HIV-monoinfected	HIV/HCV-coinfected	p-value
No. of subjects	1356	899	457	
Gender (male)	1087 (80.2%)	713 (79.3%)	374 (81.8%)	0.302
Age (years)	41 (15)	41 (17)	40 (11)	0.057
**Substance abuse**				
Drugs	19 (1.4%)	12 (1.3%)	7 (1.5%)	0.962
Alcohol	31 (2.3%)	18 (2%)	13 (2.8%)	0.430
Tobacco	363 (26.8%)	213 (23.7%)	150 (32.8%)	**<0.001**
Length of hospital stay (days)	15 (19)	14 (18)	15 (19)	0.978
Surgical conditions	39 (2.9%)	26 (2.9%)	13 (2.8%)	0.999
Charlson Comorbidity Index	0 (1)	1 (1)	0 (1)	0.671
**Major comorbid diseases**				
Congestive Heart Failure	72 (5.3%)	52 (5.8%)	20 (4.4%)	0.335
Stroke	32 (2.3%)	27 (3%)	5 (1.1%)	**0.045**
Deep venous thrombosis	235 (17.3%)	169 (18.8%)	66 (14.4%)	0.054
Chronic Pulmonary Disease	226 (16.6%)	125 (13.9%)	101 (22.1%)	**<0.001**
Liver Disease	169 (12.5%)	81 (9%)	88 (19.3%)	**<0.001**
Diabetes	42 (3.1%)	35 (3.9%)	7 (1.5%)	**0.027**
Renal Disease	39 (2.9%)	27 (3%)	12 (2.6%)	0.825
Cancer	167 (12.3%)	101 (11.2%)	66 (14.4%)	0.107
Metastatic Carcinoma	78 (5.7%)	50 (5.6%)	28 (6.1%)	0.765

Values were expressed as absolute number (percentage) and median (interquartile range). P-values were calculated by the Mann-Whitney *U* test and the Chi-squared test. P-values in bold indicate statistically significant differences between groups.

Abbreviations: HIV, human immunodeficiency virus; HCV, hepatitis C virus; PE, pulmonary embolism.

### Incidence of hospitalizations related to PE

**Figure 1:**
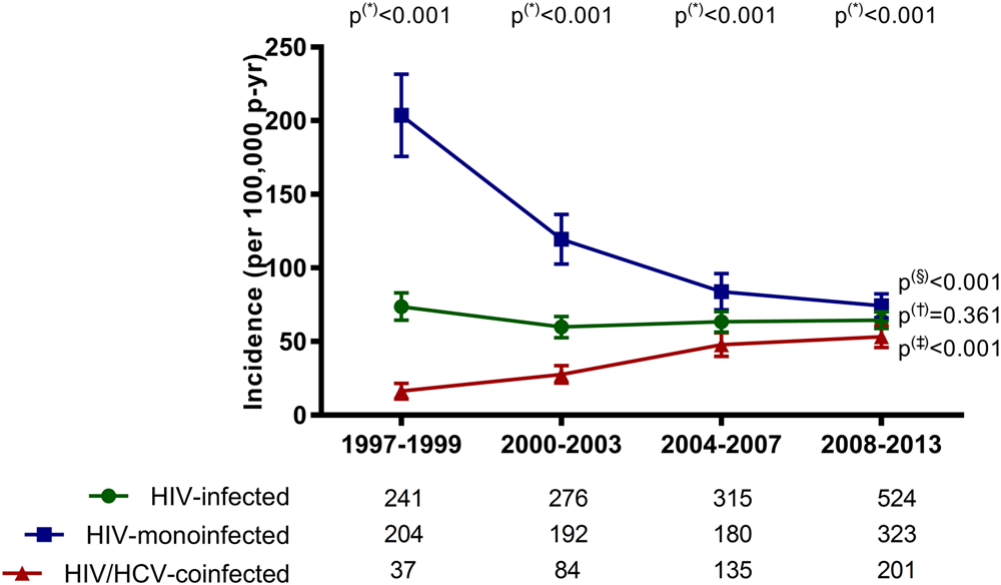
Incidence of pulmonary embolism in Spain among patients infected with HIV (1997–2013) stratified by HCV status. P-values: (*), differences calculated by the exact confidence intervals for incidence (patients monoinfected with HIV versus patients coinfected with HIV/HCV). The linear trends were estimated by the Extended Mantel Haenszel Chi-Square: (§) patients monoinfected with HIV (1997–1999 to 2008–2013); (†), all patients infected with HIV (1997–1999 to 2008–2013); (‡), patients coinfected with HIV/HCV (1997–1999 to 2008–2013). At the base of the panels, we show the total number of hospitalization in each calendar period and study group. Abbreviations: HCV, hepatitis C virus; HIV, human immunodeficiency virus.

The incidences of hospitalizations related to PE (events per 100,000 patient-years) during the four study periods in HIV-infected patients are displayed in Fig. 1 (full description in Supplementary Table 1).

Overall, the incidence of hospitalizations related to PE did not show a significant variation in all HIV-infected patients from 1997–1999 to 2008–2013 [73.7 (95% = 64.4; 83.0) vs. 64.5 (95% = 58.9; 70.0); *P* = 0.532]. When we stratified the population by HCV status, HIV-monoinfected patients had a higher rate of PE-related hospitalizations than patients coinfected with HIV/HCV throughout the complete follow-up (*P* < 0.001). However, the incidence of hospitalizations related to PE had a significant decrease in HIV-monoinfected patients (*P* < 0.001) and a significant increase in patients coinfected with HIV/HCV (*P* < 0.001). Concretely, the incidence of hospitalizations related to PE decreased significantly in patients monoinfected with HIV [from 203.6 (95%CI = 175.7; 231.6) events per 100,000 patient-years in 1997–1999 to 74.3 (95%CI = 66.1; 82.3) in 2008–2013; *P* < 0.001], while HIV/HCV-coinfected patients had a significant increase [from 16.3 (95%CI = 11; 21.6) in 1997–1999 to 53.3 (95%CI = 45.9; 60.6) in 2008–2013; *P* < 0.001].

### Mortality of PE-related hospitalizations

**Figure 2:**
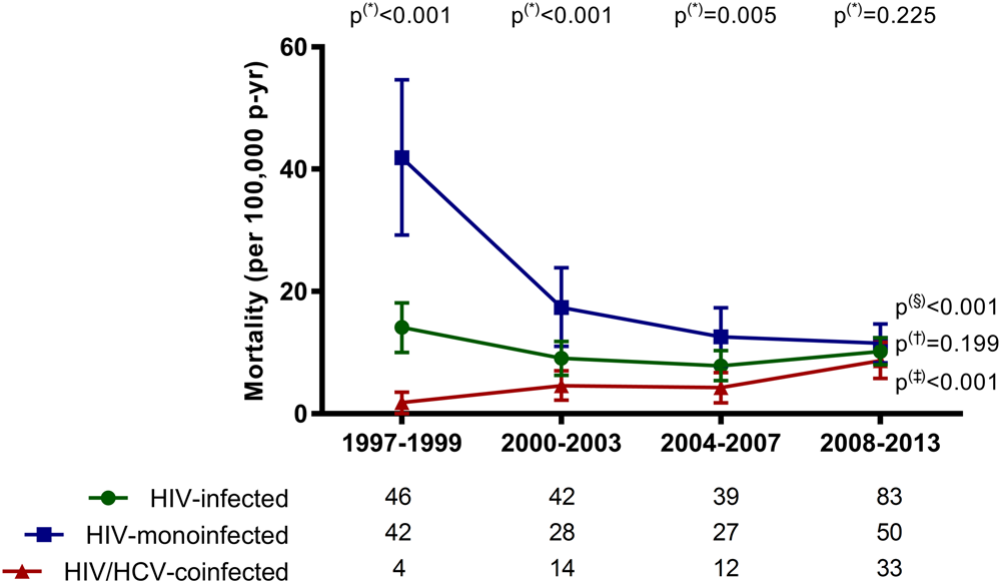
In-hospital mortality in Spain (1997–2013) among in patients infected with HIV with pulmonary embolism stratified by HCV status. P-values: (*), differences calculated by the exact confidence intervals for incidence (patients monoinfected with HIV versus patients coinfected with HIV/HCV). The linear trends were estimated by the Extended Mantel Haenszel Chi-Square: (§) patients monoinfected with HIV (1997–1999 to 2008–2013); (†), all patients infected with HIV (1997–1999 to 2008–2013); (‡), patients coinfected with HIV/HCV (1997–1999 to 2008–2013). At the base of the panels, we show the total number of hospitalization in each calendar period and study group. Abbreviations: HCV, hepatitis C virus; HIV, human immunodeficiency virus.

The mortality rate from PE-related hospitalizations (deaths per 100,000 patient-years) during the four study periods in HIV-infected patients are displayed on Fig. 2 (full description in Supplementary Table 2). The mortality rate remained stable in all HIV-infected patients (*P* = 0.199) and did not show significant differences between 1997–1999 and 2008–2013 [14.1 (95%CI = 10.0; 18.1) vs. 10.2 (95%CI = 8.0; 12.4); *P* = 0.517]. When we stratified the population by HCV status, HIV-monoinfected patients had a higher rate of mortality in PE-related hospitalizations than patients coinfected with HIV/HCV in the early calendar period (1997–1999) [41.9 (95%CI = 29.2; 54.6) vs. 1.8 (95%CI = 0; 3.5); *P* < 0.001]. Next, the mortality of PE-related hospitalizations fell sharply in patients monoinfected with HIV [from 41.9 (95%CI = 29.2; 54.6) in 1997–1999 to 17.4 (95%CI = 11; 23.9) in (2000–2003; *P* = 0.002], and maintained a plane during the late calendar periods [12.6 (95%CI = 7.8; 17.3) in 2004–2007 and 11.5 (95%CI = 8.3; 14.7) in 2008–2013]. On the contrary, the mortality rate of PE-related hospitalizations increased in patients coinfected with HIV/HCV [from 1.8 (95%CI = 0; 3.5) in 1997–1999 to 8.7 (95%CI = 5.8; 11.7) in 2008–2013; *P* = 0.002]. In the calendar period 2008–2013, patients monoinfected with HIV and patients coinfected with HIV/HCV had similar mortality rates (*P* = 0.225).

**Figure 3:**
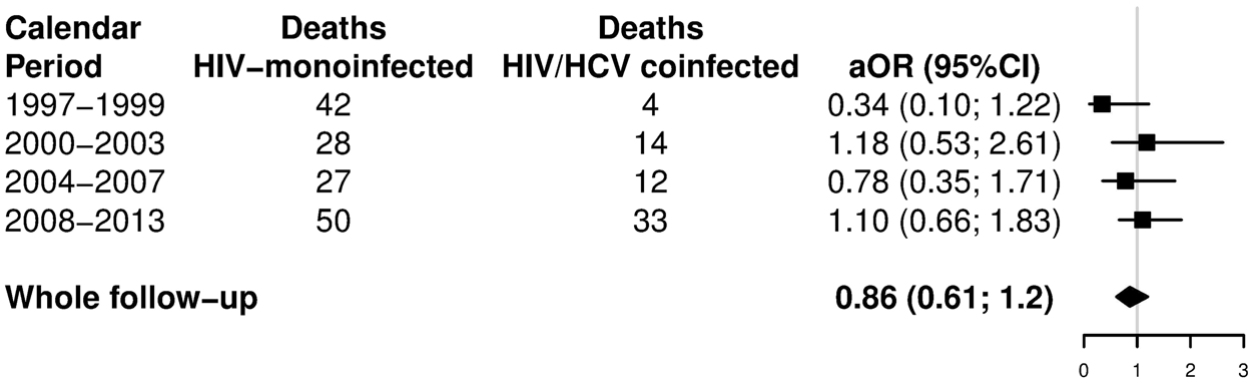
Adjusted likelihoods of death among patients infected with HIV with pulmonary embolism in Spain (1997–2013) stratified by HCV status. Abbreviations: HCV, hepatitis C virus; HIV, human immunodeficiency virus, aOR, adjusted odds ratio; 95%CI, 95% of confidence interval.

The CFR rates for PE hospitalizations related to PE are displayed in Supplementary Table 3. The trend of the CFR rate did not change during the follow-up (1997–2013) in the study groups (All HIV-infected patients, monoinfected with HIV, and coinfected with HIV/HCV). Furthermore, these three study groups showed similar CFR values. Finally, HCV coinfection was not associated with a higher odds of death when we performed an adjusted logistic regression (Fig. 3).

## Discussion

In this study, trends in both incidence and mortality of PE-related hospitalizations did not show a significant variation in all PLWH (patients monoinfected with HIV plus patients coinfected with HIV/HCV) during the entire follow-up (1997–2013). However, we found significant differences when PLWH were stratified by HCV status. Thus, both incidence and mortality of PE-related hospitalizations decreased in patients monoinfected with HIV during the follow-up, whereas both rates increased in HIV/HCV-coinfected subjects. However, patients monoinfected with HIV had a higher incidence of PE in the last cART period (2008–2013) than in patients coinfected with HIV/HCV, whereas the mortality from PE-related hospitalizations was similar in both groups. To our knowledge, our study is the first report evaluating epidemiological trends of PE-related hospitalizations in patients monoinfected with HIV stratified by their HCV serostatus.

The prothrombotic state in PLWH seems to be multifactorial^17,18^. Altered parameters related to inflammation (for example, IL-6, TNF-RI, and C-reactive protein) and coagulation (for example, tissue factor expression, FVIII, thrombin, fibrinogen, protein C, protein S, and D-dimer levels) have been found in these patients both without antiretroviral therapy and while on cART^17,19,20^. However, cART may improve the hypercoagulable state, but does not normalize most parameters^21^. Moreover, HCV coinfection and severity of liver disease are related to an increased prothrombotic state, since elevated levels of inflammation and coagulation have been described in HIV/HCV-coinfected patients^22–24^. Although there are fewer data in patients coinfected with HIV/HCV, there also appears to be a reduction in the prothrombotic state after achieving sustained virological response with HCV antiviral therapy^25,26^.

Our study shows that the epidemiological trends of PE in PLWH were different from the data of the general population in Spain^8^. In a recent article made with data from the Spanish MBDS, Miguel-Dıez *et al*.^8^ found that the PE incidence increased from 20.4 to 32.7 hospitalizations per 100,000 person-years in Spain and the mean in-hospital mortality decreased from 12.9% in 2002 to 8.32% in 2011. Another remarkable finding in our study was that the incidence, mortality, and CFR in PLWH (patients monoinfected with HIV plus patients coinfected with HIV/HCV) did not change during the follow-up. However, the incidence of hospitalizations related to PE was higher than in HIV seronegative patients in Spain, whereas CFR values were only slightly higher in HIV-infected patients^8^.

In our study, HIV-monoinfected patients showed a significant decrease both in incidence and mortality for PE-related hospitalizations. The increasing use of cART may have influenced these findings. The percentage of PLWH on cART increased significantly in Spain during the last decade^27^, increasing the percentage of patients with undetectable HIV viral load and higher CD4+ T-cell counts^27^, and decreasing their risk of cardiovascular events^28^. Furthermore, the enhancement in preventive interventions to reduce the cardiovascular risk in PLWH on cART has decreased the mortality related to cardiovascular disease in high-income countries^3^.

In this study, patients coinfected with HIV/HCV had a reverse temporal trend of both incidence and mortality for PE-related hospitalizations compared to patients monoinfected with HIV. In the calendar period 1997–1999, the values of incidence and mortality of hospitalizations related to PE was far lower in patients coinfected with HIV/HCV than in patients monoinfected with HIV; but both rates increased significantly in patients coinfected with HIV/HCV in the following years. In the last calendar period (2008–2013), the PE incidence related to hospitalizations was lower in patients coinfected with HIV/HCV than in patients monoinfected with HIV, but the mortality of PE-related hospitalizations was similar for both groups. The explanation for these epidemiological trends is not simple, especially if we consider that our data source was the MBDS. On the one hand, over the study period, HCV screening might have been different in patients admitted to hospitals with a PE diagnosis (e.g., more HCV testing in the later periods than during earlier periods in HIV-positive patients), but HCV screening in PLWH has not changed significantly in Spain over the study period (around 95%)^2,29^. On the other hand, the death could have been an important competing risk for PE in patients coinfected with HIV/HCV during all follow-up, since the mortality trend for HIV-infected subjects declined significantly in Spain, primarily at the expense of patients monoinfected with HIV, while mortality among patients coinfected with HIV/HCV did not decline^30^. Additionally, the primary mode of HIV acquisition in patients infected with HCV was the injection drug use^2,27,29^, and these subjects had a high risk of death^2^. In fact, intravenous drug use in HIV-infected patients is a significant factor for right-sided endocarditis and PE^31^. However, we did not have data on active or recent intravenous drug use to evaluate its influence on PE trends and the real impact of chronic hepatitis C. Also note that more efficacious and safer cART regimens^32^ and an increasing treatment uptake of anti-HCV therapy^2^ in patients coinfected with HIV/HCV have been associated with mortality reduction in these patients^6,33–35^.

## Limitations

Finally, several aspects must be considered for the correct interpretation of the results.

Firstly, we did not have figures for the global number of PLWH in Spain from 1997 to 2013, since there was no data of national coverage of HIV diagnoses in our country during this period. Instead, we used PLWH estimation data from the National Centre of Epidemiology (NCE, Instituto de Salud Carlos III, Madrid, Spain)^36^. However, NCE did not provide data of gender, age or comorbidities and we could not calculate the rates stratified by the main cardiovascular risk factors (gender, age, diabetes, hypertension, etc.). Secondly, it was not known how many patients with thrombosis did not need any hospital admission for systemic anticoagulation or how many patients died before hospital admission, since the MBDS only provides information about hospitalized patients. Additionally, the hospitalization may be avoided in many patients with thrombosis due to the development of better drugs for outpatient treatment^37^. This fact may be more likely to occur for patients monoinfected with HIV than patients coinfected with HIV/HCV because coinfection with HCV leads to more frequent liver disease that requires hospitalization. Thirdly, we did not have access to critical clinical and epidemiological data related to HIV infection and PE management, which could have helped us to interpret our results more thoroughly. Fourthly, the presence of anti-HCV antibodies defined the HCV status because we did not have any data about active HCV infection (HCV-RNA in serum or plasma), which creates an overestimation of the number of HCV-infected patients. Finally, we could not assess the impact of HCV therapy on the PE trends in patients coinfected with HIV/HCV.

## Conclusions

In conclusion, the epidemiological trends of PE in PLWH changed during the cART era, with decreasing values of both incidence and mortality in patients monoinfected with HIV and increases of both rates in patients coinfected with HIV/HCV. Further studies are needed to investigate the impact of lifestyle, active HCV infection, and HCV clearance following HCV therapy.

## Materials and Methods

### Study population

We performed a retrospective study of all subjects older than 16 years who were discharged with a PE diagnosis from Spanish hospitals from January 1, 1997, to December 31, 2013. The study period was divided into four calendar periods^38^: (a) 1997–1999; (b) 2000–2003; (c) 2004–2007; and (d) 2008–2013.

Data of patients were collected from the Minimum Basic Data Set (MBDS) of the Ministry of Health, Consumption and Social Welfare (MHCSW). Data were collected in 2016 and analyzed in 2017. The MBDS contains clinical and epidemiological data recorded at the hospital discharge. The MDBS has 92% coverage of all Spanish hospitals [public hospitals (84.14%) and private hospitals (15.86%)]^39^. Additionally, 100% of the Spanish population has free medical care provided by the National Health System (MHCSW).

The MBDS included 14 discharge diagnoses and 20 procedures performed during the hospital stay according to the *International Classification of Diseases, 9th ed, Clinical Modification* (ICD-9-CM). Furthermore, the MBDS provided the date of birth, sex, dates of hospital admission and discharge, and outcome at discharge. The Spanish MHCSW has established standards for record keeping and conducts periodic audits.

### Ethics statement

The MHCSW requested hospitals the health-related personal data, which were added to Spanish MBDS according to Spanish legislation^40^. The MHCSW approved our study and the data were treated with full confidentiality and. The signed patient’s consent was not needed since MBDS is an anonymous dataset and has a mandatory nature. Moreover, the Research Ethics Committee (Comité de Ética de la Investigación y de Bienestar Animal; CEI PI 69_2012) of the Instituto de Salud Carlos III (Madrid, Spain) approved our study.

### Study groups

Viral infection status was defined by ICD-9-CM codes (see Supplementary Table 4): (i) HIV infection (042 or V08); (ii) HCV infection (070.41, 070.44, 070.51, 070.54, 070.7x, or V02.62); (iii) HBV infection (070.2x, 070.3x, or V02.61). Patients with HBV infection were excluded. From these ICD-9-CM codes, three groups were established: (i) HIV-infected (all patients infected with HIV); (ii) HIV-monoinfected (only HIV); (iii) HIV/HCV-coinfected (both HIV and HCV). We did not have any information about if a person had the diagnosis of HIV infection and HCV infection previously, concomitantly to, or after the diagnosis of PE.

### Outcome variables

We selected subjects with PE diagnosis codes [415.11 and 415.19 (see Supplementary Table 4)] in the MBDS, according to the criteria of Miguel-Diez *et al*.^8^. The PE diagnosis was performed according to the standard radiological procedure in each hospital of the Spanish National Health System by using computed tomography pulmonary angiography, pulmonary scintigraphy, or others. The diagnostic code for PE was assigned by physicians when the diagnostic imaging was positive (see examples in Supplementary Figure 1). A discharge record with a PE diagnosis in the MBDS was defined a PE-related hospitalization. The first hospital discharge with PE diagnosis was defined as the index episode. Patients readmitted with a later PE event (included in any position) were not identified as new episodes of PE. We analyzed the following outcome variables: i) new PE diagnosis (incidence); ii) death among patients with PE diagnosis (in-hospital PE mortality). We did not have any information about if a person died in the days or few weeks following discharge, which could have also been attributed to PE.

### Reference populations

The NCE provided the estimation of the number of PLWH in Spain (see Supplementary Table 5)^38^. We also estimated the number of people monoinfected with HIV and HIV/HCV-coinfected in Spain using data from the hospital survey of HIV/AIDS patients coordinated by the NCE^27^, and the reports of the “Grupo de Estudio de Sida” (GeSIDA)^41^ and the “Asociación Médica VACH de Estudios Multicéntricos (AMVACH)”^42^.

### Statistical analysis

We calculated the PE incidence (new PE cases per 100,000 patient-years) and PE mortality (in-hospital PE-related deaths per 100,000 patient-years) as the ratio between the number of events and the number of persons at risk within each calendar period, according to reference populations (see the previous section). The case fatality rate (CFR) was the ratio between the number of patients with PE-related deaths and the number of patients with a PE-related hospitalization (percentage, %).

The chi-squared test and Fisher’s exact test were used to analyze categorical data and proportions, as was required. Continuous variables were studied using the T-Test or Mann-Whitney U test. The Extended Mantel Haenszel Chi-Square was used for evaluating the temporal trends of rates. Multivariate logistic regressions were used to calculate the odds for in-hospital PE-related death adjusted by age, sex, tobacco usage, and Charlson co-morbidity index (CCI).

The R statistical package version 3.1.1 (R Foundation for Statistical Computing, Vienna, Austria) was used to perform the statistical analysis. All the statistical tests were considered significant with values of p < 0.05 (two-tailed).

## Electronic supplementary material

Supplementary Information
