# Supplementary material for: Trends in pulmonary embolism in patients infected with HIV during the combination antiretroviral therapy era in Spain: A nationwide population-based study
Source: Sci Rep. 2018 Aug 14;8:12137. doi: 10.1038/s41598-018-29739-2 (PMC6092411; doi:10.1038/s41598-018-29739-2)
Supplement: Supplementary file 1 — Supplementary Information [file 41598_2018_29739_MOESM1_ESM.pdf]

Title page

Title: Trends in pulmonary embolism in patients infected with HIV during the combination antiretroviral therapy era in Spain: A nationwide population-based study

Running Head: Pulmonary embolism and HIV infection

Authors: Alejandro ALVARO-MECA, Ph.D. [1]; Pablo RYAN, M.D., Ph.D. [2]; Dariela MICHELOUD, M.D., Ph.D. [3]; Angel GIL DE MIGUEL, M.D., Ph.D. [1]; Juan BERENGUER, M.D., Ph.D. [4,5]; Salvador RESINO, Ph.D. [6]

Current affiliation: (1) Departamento de Medicina Preventiva y Salud Pública, Facultad de Ciencias de la Salud, Universidad Rey Juan Carlos, Alcorcón, Madrid, Spain. (2) Servicio de Medicina Interna, Hospital Universitario Infanta Leonor, Madrid, Spain. (3) Servicio de Urgencias, Hospital General Universitario "Gregorio Marañón," Madrid, Spain. (4) Unidad de Enfermedades Infecciosas/VIH; Hospital General Universitario "Gregorio Marañón", Madrid, Spain. (5) Instituto de Investigación Sanitaria Gregorio Marañón (IiSGM), Madrid, Spain. (6) Unidad de Infección Viral e Inmunidad, Centro Nacional de Microbiología, Instituto de Salud Carlos III, Majadahonda, Madrid, Spain.

Corresponding author: Salvador Resino; Centro Nacional de Microbiología, Instituto de Salud Carlos III (Campus Majadahonda); Carretera Majadahonda-Pozuelo, Km 2.2; 28220 Majadahonda (Madrid); e-mail: sresino@isciii.es

Supplementary Table 1. Epidemiological incidence trends of PE-related hospitalizations (events per 100,000 patients/year) in Spain (1997 to 2013) stratified by HIV and HCV infection status.

	**HIV-infected**		**HIV-monoinfected**		**HIV/HCV-coinfected**		
	**No.**	**Rate (95%CI)**	**No.**	**Rate (95%CI)**	**No.**	**Rate (95%CI)**	**p-value** (*)
Whole follow-up	1356	64.5 (61.1; 68.0)	899	98.7 (92.2; 105.1)	457	38.4 (34.9; 41.9)	**<0.001**
1997-1999	241	73.7 (64.4; 83.0)	204	203.6 (175.7; 231.6)	37	16.3 (11; 21.6)	**<0.001**
2000-2003	276	59.9 (52.5; 67.0)	192	119.5 (102.6; 136.4)	84	27.7 (21.8; 33.6)	**<0.001**
2004-2007	315	63.4 (56.4; 70.4)	180	83.8 (71.6; 96.1)	135	47.8 (39.8; 55.9)	**<0.001**
2008-2013	524	64.5 (58.9; 70.0)	323	74.2 (66.1; 82.3)	201	53.3 (45.9; 60.6)	**<0.001**
**P-values** (*)							
Differences: 97-99 vs. 00-03		0.096		**<0.001**		**0.035**	
Differences: 97-99 vs. 04-07		0.480		**<0.001**		**<0.001**	
Differences: 97-99 vs. 08-13		0.532		**<0.001**		**<0.001**	
Differences: 00-03 vs. 04-07		0.999		**0.004**		**<0.001**	
Differences: 00-03 vs. 08-13		0.999		**<0.001**		**<0.001**	
Differences: 04-07 vs. 08-13		0.999		0.999		0.999	
**P-values** (§)							
Linear trend		0.361		**<0.001**		**<0.001**	

Values are expressed as absolute count; and rate (95% confidence interval (95% CI)).
P-values: (*), differences by the exact confidence intervals for incidence; (§), linear trend from 1997-1999 to 2008-2013 by the Extended Mantel Haenszel Chi Square. Statistical significant differences are shown in bold.
Abbreviations: HCV, hepatitis C virus; HIV, human immunodeficiency virus; PE, pulmonary embolism.

Supplementary Table 2. Epidemiological mortality trends of PE-related hospitalizations (events per 100,000 patients/year) in Spain (1997 to 2013) stratified by HIV and HCV infection status.

	**HIV-infected**		**HIV-monoinfected**		**HIV/HCV-coinfected**		
	**No.**	**Rate (95%CI)**	**No.**	**Rate (95%CI)**	**No.**	**Rate (95%CI)**	**p-value** (*)
Whole follow-up	210	10.0 (8.6; 11.3)	147	16.1 (13.5; 18.7)	63	5.3 (4; 6.6)	**<0.001**
1997-1999	46	14.1 (10.0; 18.1)	42	41.9 (29.2; 54.6)	4	1.8 (0; 3.5)	**<0.001**
2000-2003	42	9.1 (6.3; 11.8)	28	17.4 (11; 23.9)	14	4.6 (2.2; 7)	**<0.001**
2004-2007	39	7.8 (5.4; 10.3)	27	12.6 (7.8; 17.3)	12	4.3 (1.8; 6.7)	**0.001**
2008-2013	83	10.2 (8.0; 12.4)	50	11.5 (8.3; 14.7)	33	8.7 (5.8; 11.7)	0.225
**P-values** (*)							
Differences: 97-99 vs. 00-03		0.241		**0.002**		0.473	
Differences: 97-99 vs. 04-07		**0.045**		**<0.001**		0.726	
Differences: 97-99 vs. 08-13		0.517		**<0.001**		**0.002**	
Differences: 00-03 vs. 04-07		0.999		0.999		0.999	
Differences: 00-03 vs. 08-13		0.999		0.498		0.248	
Differences: 04-07 vs. 08-13		0.999		0.999		0.165	
**P-values** (§)							
Linear trend		0.199		**<0.001**		**<0.001**	

Values are expressed as absolute count; and rate (95% confidence interval (95% CI)).
P-values: (*), differences by the exact confidence intervals for incidence; (§), linear trend from 1997-1999 to 2008-2013 by the Extended Mantel Haenszel Chi Square. Statistical significant differences are shown in bold.
Abbreviations: HCV, hepatitis C virus; HIV, human immunodeficiency virus; PE, pulmonary embolism.

Supplementary Table 3. Epidemiological trends of case fatality rate of PE-related hospitalizations (%) in Spain (1997 to 2013) stratified by HIV and HCV infection status.

	**HIV-infected**		**HIV-monoinfected**		**HIV/HCV-coinfected**		
	**No.**	**Rate (95%CI)**	**No.**	**Rate (95%CI)**	**No.**	**Rate (95%CI)**	**p-value** (*)
Whole follow-up	210	15.49 (13.62; 17.55)	147	16.35 (14.03; 18.97)	63	13.79 (10.83; 17.37)	0.248
1997-1999	46	19.09 (14.44; 24.74)	42	20.59 (15.39; 26.92)	4	10.81 (3.52; 26.36)	0.244
2000-2003	42	15.22 (11.3; 20.13)	28	14.58 (10.07; 20.56)	14	16.67 (9.73; 26.73)	0.794
2004-2007	39	12.38 (9.05; 16.65)	27	15 (10.28; 21.25)	12	8.89 (4.88; 15.34)	0.145
2008-2013	83	15.84 (12.88; 19.32)	50	15.48 (11.81; 20)	33	16.42 (11.72; 22.43)	0.871
**P-values** (*)							
Differences: 97-99 vs. 00-03		0.880		0.455		0.999	
Differences: 97-99 vs. 04-07		0.119		0.591		0.999	
Differences: 97-99 vs. 08-13		0.937		0.496		0.999	
Differences: 00-03 vs. 04-07		0.999		0.999		0.389	
Differences: 00-03 vs. 08-13		0.999		0.999		0.999	
Differences: 04-07 vs. 08-13		0.607		0.999		0.205	
**P-values** (§)							
Linear trend		0.367		0.242		0.510	

Values are expressed as absolute count; and rate (95% confidence interval (95% CI)).
P-values: (*), differences by the exact confidence intervals for incidence; (§), linear trend from 1997-1999 to 2008-2013 by the Extended Mantel Haenszel Chi Square. Statistical significant differences are shown in bold.
Abbreviations: HCV, hepatitis C virus; HIV, human immunodeficiency virus; PE, pulmonary embolism

Supplementary Table 4. Summary of ICD-9-CM coding used for baseline comorbidities investigated in this study.

Description	ICD-9-CM
**Viral infection status**	
HIV infection	042 or V08
HCV infection	070.44, 070.54, 070.7x, or V02.62
HBV infection	070.2x, 070.3x, or V02.61
**Conditions influencing in health status**	
Surgical conditions	V42, V45
Trauma	E880* to E929*, E950 to E999*
**Comorbid diseases (Charlson index)**	
Myocardial Infarction	410, 412
Congestive Heart Failure	398.91, 402.01, 402.11, 402.91, 404.01, 404.03, 404.11, 404.13, 404.91, 404.93, 425.4, 425.5, 425.7, 425.8, 425.9, 428
Peripheral Vascular Disease	093.0, 437.3, 440, 441, 443.1, 443.2, 443.8, 443.9, 447.1, 557.1, 557.9, V434
Cerebrovascular Disease	362.34, 430, 431, 432, 433, 434, 435, 436, 437, 438
Chronic Pulmonary Disease	416.8, 416.9, 490, 491, 492, 493, 494, 495, 496, 500, 501, 502, 503, 504, 505, 5064, 508.1, 508.8
Connective Tissue Disease-Rheumatic Disease	446.5, 710.0, 710.1, 710.2, 710.3, 710.4, 714.0, 714.1, 714.2, 714.8, 725
Mild Liver Disease	070.22, 070.23, 070.32, 070.33, 070.44, 070.54, 070.6, 070.9, 570, 571, 573.3, 573.4, 573.8, 573.9, V427
Moderate or Severe Liver Disease	456.0, 456.1, 456.2, 572.2, 572.3, 572.4, 572.8
Diabetes without complications	250.0, 250.1, 250.2, 250.3, 250.8, 250.9
Diabetes with complications	250.4, 250.5, 250.6, 250.7
Renal Disease	403.01, 403.11, 403.91, 404.02, 404.03, 404.12, 404.13, 404.92, 404.93, 582, 583.0, 583.1, 583.2, 583.4, 583.6, 583.7, 585, 586, 588.0, V420, V451, V56
Cancer	140, 141, 142, 143, 144, 145, 146, 147, 148, 149, 150, 151, 152, 153, 154, 155, 156, 157, 158, 159, 160, 161, 162, 163, 164, 165, 170, 171, 172, 174, 175, 176, 179, 180, 181, 182, 183, 184, 185, 186, 187, 188, 189, 190, 191, 192, 193, 194, 195, 200, 201, 202, 203, 204, 205, 206, 207, 208, 238.6
Metastatic Carcinoma	196, 197, 198, 199
**Abuse of alcohol and drugs**	
Abuse of drugs	304.0,304.1,304.2,304.3,304.4,304.5,304.6,304.7,304.8,304.9,305.2,305.3,305.4,305.5,305.6,305.7,305.8,305.9, 292.0,969.6,965.01,292.82,292.83,292.84,292.89,292.9,292.11,292.12,292.2,648.3
Abuse of alcohol	305.0,303.0,303.9,291.0,291.1,291.2,291.3,291.4,291.5,291.8,291.9,571.0,571.1,571.2,571.3,425.5,535.3,357.5, 265.2, V11.3,790.3,980.0
Abuse of tobacco	305.1,V158.2
**Venous thromboembolism**	
Pulmonary embolism	415.11, 415.19
Deep venous thrombosis	453.4x, 453.8x

Supplementary Figure 1. Cross-sectional images of computed tomography (CT) used to diagnosis the acute pulmonary embolism.
Images A and B: a 35-year-old man infected with HIV with bilateral pulmonary thromboembolism with filling defects in the right main pulmonary artery and interlobar artery.
Images C and D: a 39-year-old woman co-infected with HIV and HCV who presents multiple filling defects in the pulmonary arterial tree bilaterally and signs of right ventricular overload in the CT scan.

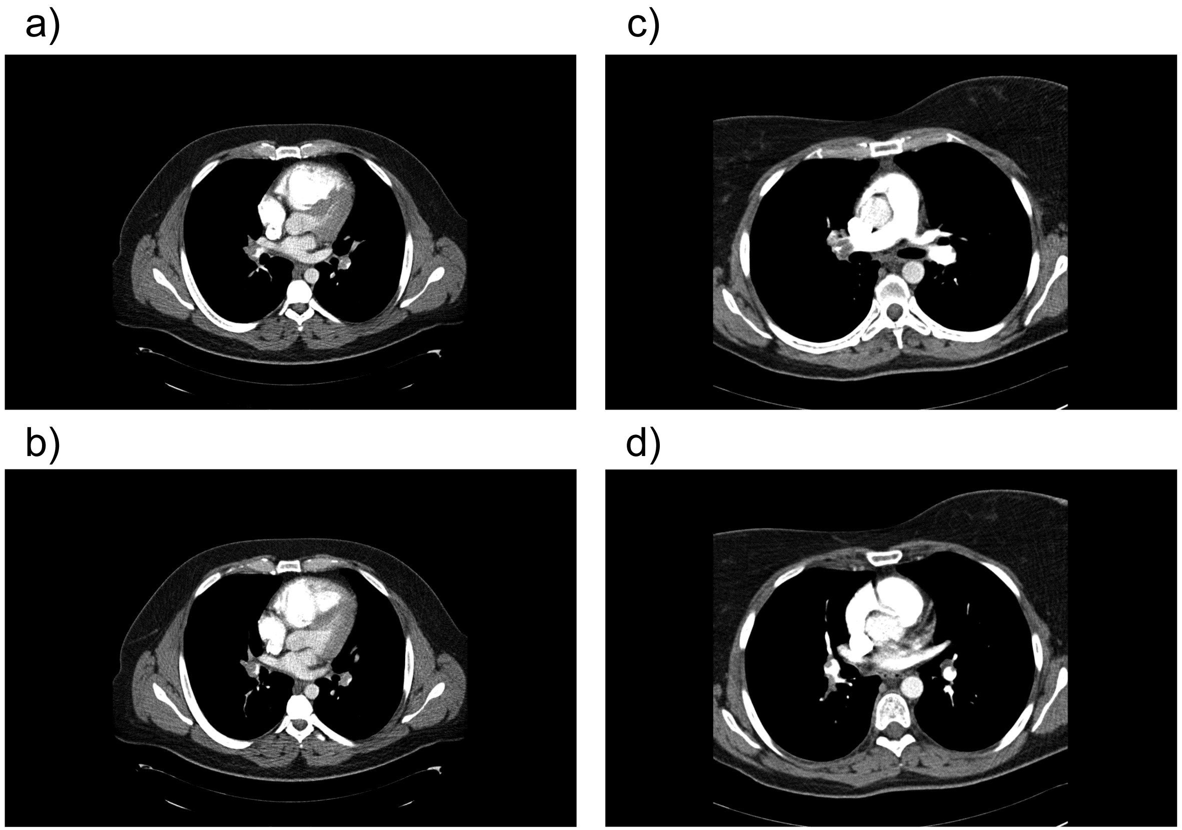

Supplementary Table 5. Estimation of the number of people over 15 years of age coinfected with HIV and HCV in Spain.

Year	HIV-infected patients (No.) (*)	HCV-positive antibody (%) (‡)	HIV/HCV-coinfected patients (%) (†)	HIV/HCV-coinfected patients (No.) (¥)	HIV-monoinfected (No.) (§)
**1997**	106,483	69	69.8	74,353	32,130
**1998**	109,419	69	69.4	75,962	33,457
**1999**	111,281	69	68.9	76,689	34,592
**2000**	113,130	69	68.3	77,274	35,856
**2001**	114,824	69	66.7	76,605	38,219
**2002**	116,766	65	64.4	75,286	41,480
**2003**	118,964	-	62.1	73,905	45,059
**2004**	121,038	57.1	59.9	72,524	48,514
**2005**	123,069	58.5	57.7	71,113	51,956
**2006**	125,257	53.1	55.7	69,882	55,375
**2007**	127,615	50.8	53.8	68,719	58,896
**2008**	129,765	48.7	51.6	67,018	62,747
**2009**	131,888	47.7	49.2	64,992	66,896
**2010**	134,392	45.7	47.1	63,409	70,983
**2011**	136,747	42.6	45.4	62,126	74,621
**2012**	138,978	42.8	43.6	60,723	78,255
**2013**	141,052	42.6	41.9	59,163	81,889

(*), The estimation of number of people living with HIV/AIDS in Spain was provided by the National Centre of Epidemiology (Instituto de Salud Carlos III, Madrid, Spain). This estimation was done using the Estimation and Projection Package (EPP) and Spectrum software, two programs developed by the Joint UNAIDS/WHO for estimating and projecting HIV prevalence at country level [1, 2].

(‡), The percentage of patients with HCV antibodies was collected from the "Asociación Médica VACH de Estudios Multicentricos (AMVACH)" (1999-2001) [3], the "Grupo de Estudio de Sida" (GeSIDA) (2002) [4], and the "Hospital survey of patients infected with HIV", a second-generation surveillance system in people living with HIV coordinated by the National Centre of Epidemiology (2004-2013) [5, 6].

(†), The final estimation of the percentage of subjects coinfected with HIV and HCV in Spain was obtained from a regression model for imputing missing values and smoothing the numbers according to the temporal trend of the data.

(¥), The estimation of the number of subjects coinfected with HIV and HCV in Spain was the result of multiplying the number of individuals infected with HIV and the percentage of patients coinfected with HIV and HCV.

(§), The estimation of the number of HIV-monoinfected patients in Spain was the result of subtracting the number of individuals infected with HIV and the number of patients coinfected with HIV and HCV.
